# Factors associated with utilization of community health workers in improving access to malaria treatment among children in Kenya

**DOI:** 10.1186/1475-2875-11-S1-P142

**Published:** 2012-11-09

**Authors:** James Kisia, Florence Nelima, David Odhiambo Otieno, Kioko Kiilu, Emmanuel Wamalwa, Salim Sohani, Kendra Siekmans, Andrew Nyandigisi, Willis Akhwale

**Affiliations:** 1 Kenya Red Cross Society, P.O Box 40712, Nairobi 00100, Kenya; 2 Ministry of Public Health and Sanitation, Kenya; 3 University of Nairobi Institute of Tropical and Infectious Community Diseases (UNITID), P.O Box 30197, Nairobi 00100, Kenya; 4 Canadian Red Cross, 170 Metcalfe Street, Suite 300, Ottawa, ON K2P 2P2, Canada; 5 HealthBridge, 1004-1 Nicholas Street, Ottawa, ON K1N 7B7, Canada; 6 Care International in Kenya, P.O. Box 43864, Nairobi 00100, Kenya

## Background information

The success of community case management in improving access to effective malaria treatment for young children relies on broad utilization of community health workers (CHWs) to diagnose and treat fever cases. A better understanding of the factors associated with CHW utilization is crucial in informing national malaria control policy and strategy in Kenya. Specifically, little is known in Kenya on the extent to which CHWs are utilized, the characteristics of families who report utilizing CHWs and whether utilization is associated with improved access to prompt and effective malaria treatment. This paper examines factors associated with utilization of CHWs in improving access to malaria treatment among children under five years of age by women caregivers in two malaria endemic districts in Kenya

## Methods

This study was conducted in 113 hard-to-reach and poor villages in Malindi and Lamu districts in the coastal region classified as having endemic transmission of malaria. A cross-sectional household survey was conducted using a standardized malaria indicator questionnaire at baseline (n = 1,187) and one year later at end-line assessment (n = 1,374) using two-stage cluster sampling.

## Results

There was an increase in reported utilization of CHWs as source of advice/treatment for child fevers from 2% at baseline to 35% at end-line, accompanied by a decline in care-seeking from government facilities (from 67% to 48%) and other sources (26% to 2%) including shops. The most poor households and poor households reported higher utilization of CHWs at 39.4% and 37.9% respectively, compared to the least poor households (17.0%). Households in villages with less than 200 households reported higher CHWs utilization as compared to households in villages having >200 households. Prompt access to timely and effective treatment was 5.7 times higher (95% CI 3.4-9.7) when CHWs were the source of care sought. Adherence was high regardless of whether source was CHWs (73.1%) or public health facility (66.7%).

**Table 1:** 

Characteristic	BASELINE	ENDLINE	P-Values
Sample size (N)	269	345	
Women caregiver education level			0.125
None	53.2 (143)	57.7 (199)	
Primary	43.5 (117)	41.2 (142)	
Secondary	3.4 (9)	1.2 (4)	
Woman caregiver age category			0.332
<=20 y	21.2 (57)	15.7 (54)	
21-30 y	44.6 (120)	47.1 (162)	
31-50 y	26.7 (72)	29.4 (101)	
51+ y		1.1 (4)	
Unknown	7.4 (20)	6.7 (23)	
Male household head	84.1 (216)	81.7 (282)	0.285
Household owns radio	40.9 (105)	32.8 (113)	0.041
Household owns bicycle	56.4 (145)	48.1 (166)	0.044
Household owns mosquito nets	81.3 (209)	89.3 (308)	0.006
Village size			0.531
<= 60 households	21.9 (59)	18.6 (64)	
61 to 100 households	24.5 (66)	27.8 (96)	
101 to 200 households	31.6 (85)	32.2 (111)	
>200 households	21.9 (59)	21.5 (74)	
Household wealth rank			0.335
Most poor	22.3 (60)	23.8 (82)	
Poor	54.3 (146)	57.7 (199)	
Least poor	23.4 (63)	18.6 (64)	

**Table 2:** Source of advice/treatment for children with fever in past 2 weeks among those who reported seeking advice/treatment

Source of advice/treatment	Baseline (N=235)		Endline (N=298)^1^	
	
	n	%	n	%
CHW/Red Cross volunteer	5	2.1	103	34.6
Government health facilities	157	66.8	143	48.0
Private medical sector^1^	12	5.1	46	15.4
Other sources^1^	59	26.0	6	2.0

**Table 3:** Women caregiver, household and village characteristics associated with utilization of CHW services for child fever advice/treatment

Characteristic	CHW (N=103)	Other (N=195)	p-value
Women caregiver education			
No formal education	57.3 (59)	56.4 (110)	0.885
Primary/secondary	42.7 (44)	43.6 (85)	

Women caregiver age group	N=98	N=178	
≤20 y	16.3 (16)	17.4 (31)	0.993
21-30 y	50.0 (49)	48.9 (87)	
31-40 y	29.6 (29)	29.2 (52)	
41+ y	4.1 (4)	4.5 (8)	

Attended ANC during last pregnancy	N=100 69.0 (69)	N=193 65.8 (127)	0.581

IPT (2+ doses SP) during last pregnancy	N=56 82.1 (46)	N=111 84.7 (94)	0.674

Knowledge of AL as new anti-malarial drug	44.7 (46)	N=194 29.9 (58)	0.010

Identified sleeping under net as way to prevent malaria	84.5 (87)	78.0 (152)	0.179

Household wealth rank Most poor Poor Least poor	27.2 (28) 64.1 (66) 8.7 (9)	22.1 (43) 55.4 (108) 22.6 (44)	0.012

Household size	N=102	N=194	
2 to 4	22.6 (23)	24.7 (48)	0.425
5 to 7	41.2 (42)	46.4 (90)	
8 or more	36.3 (37)	28.9 (56)	

Village size <60 households	22.3 (23)	17.4 (34)	0.008
61-100 households	33.0 (34)	24.6 (48)	
101-200 households	34.0 (35)	30.3 (59)	
>200 households	10.7 (11)	27.7 (54)	

Visited by CHW in past 3 months	94.2 (97)	28.7 (56)	<0.001

**Table 4:** A cross tabulation of timely provision of AL and source of the antimalarial

Timing of AL treatment	CHW as source of advice/treatment		p-value
		
	Yes	No	
AL given within 24 hours	57.3 (59)	19.0 (37)	<0.001
AL given within 48 hours	79.6 (82)	36.4 (71)	<0.001
AL given at any time	90.3 (93)	45.1 (88)	<0.001

## Conclusion

The results of this study provide evidence that use of trained and supervised community health workers in community case management improved management of uncomplicated child fever cases in hard to reach villages in Malindi and Lamu District in Coastal Province of Kenya. In addition to this, poverty seems to be closely linked to child caregivers seeking services of community-based service providers, highlighting the impediment of poverty towards accessibility of cost sharing services widely practiced in Kenyan public health facilities. Policy actions to address barriers to effective utilization of CHWs in healthcare delivery should be scaled up in such hard to reach communities. The government and partners should, therefore, invest more in mechanisms which support CHW utilization especially the roll out of the Community Health Strategy 2006 as part of successful control of malaria and other infectious diseases.

The potential for utilization of CHWs in improving access to malaria treatment at the community level is promising. This will not only enhance access to treatment by the poorest households but also provide early and appropriate treatment to vulnerable individuals, especially those living in hard to reach areas**.**

